# Heavy metal contents and enzymatic activity in soils exposed to the impact of road traffic

**DOI:** 10.1038/s41598-019-56418-7

**Published:** 2019-12-27

**Authors:** Hanna Jaworska, Joanna Lemanowicz

**Affiliations:** UTP University of Sciences and Technology, Faculty of Agriculture and Biotechnology Laboratory of Soil Science and Biochemistry, Bydgoszcz. 85-029 Bydgoszcz, Bernardyńska 6, Wrocław, Poland

**Keywords:** Environmental impact, Natural hazards

## Abstract

The aim of the research was to evaluate the influence of car traffic on the content of selected heavy metals in soil from a park area, and to define the dependency between their content and enzyme activity. Soil samples were collected from 13 points located along a communication route, each 100 m from the next and 50 m from the border of the road. Soil material was obtained from two depths (0–20 cm and 20–40 cm) and analysed for: pH in H_2_O and in KCl, OC, and texture by laser method. Total content of heavy metals (Pb, Cd, Zn, Cu, Ni), available phosphorus and the activity of selected enzymes: catalase, dehydrogenase, acidic and alkaline phosphatase were all determined. The examined soils have the texture of loamy sands or sands (USDA 2012), slightly acidic or neutral pH, Organic Carbon (OC) content in the range from 3.50 to 13.80 g kg^−1^. The total contents of elements in surface horizons were, in order of decreasing concentrations: Pb>Zn>Cu>Ni>Cd, although in subsurface horizons it was Zn>Pb>Cu>Ni>Cd. Contamination Factor (CF) determined for Ni, Pb, Cd, Zn, Cu reaches higher values in samples from subsurface horizons, which confirms the influence of car traffic on the content of heavy metals in the surrounding soils. The calculated CF shows contamination is moderate for Ni, Cd, Zn and Cu and high for Pb and Cu. The investigated soils may be classified as class IV (low) in terms of available phosphorus. The activity of the examined enzymes was higher in soil samples collected from the 0–20 cm layer than from 20–40 cm. The correlation analysis indicates a significant positive dependency between OC content in soils and enzymatic activity. Principal Component Analysis (PCA) was also performed. Two principal components PC1 and PC2 account for 66.57% of the variability.

## Introduction

Exhaust fume emissions are one of the main pollutants in areas adjacent to highways. The increase day-to-day car transportation in developing countries has increased pollution of the natural environment. This source contributes to the emission of air pollutants, including: heavy metals (Cd, Zn,Cu, Pb, Ni, etc.)^1^. Increased contents of heavy metals in soils, mostly from forest areas, decrease the microbiological activity of soils and affect root systems^2,3^. Moreover, pollution by exhaust fumes is a stress factor, which may negatively affect the vitality, productivity and species composition of forests.

Street dust is a common occurrence and consists of a mixture of wear from road surface and tyres, and particulate matter emitted by road vehicle engines^4^. The content of heavy metals in soil may be affected by natural and anthropogenic factors such as road traffic^5^. Higher vehicle velocity results in increased exhaust emissions containing heavy metals, among other compounds^6^. The emission of heavy metals around roads and motorways has a limited spatial range. The accumulation is highest in soils at 20–40 m from the roadway, and is clearly lower at a distance of 100–150 m. Heavy metals contaminating the soil inhibit the growth of microorganisms, disrupting their basic physiological functions, and primarily processes related to the decomposition and transformation of organic matter. The origin of enzymes in the soil environment is mainly associated with microorganisms. These compounds are involved in synthesising proteins, carbohydrates and nucleic acids, and are involved in the circulation of carbon, nitrogen, phosphorus, sulphur and other nutrients^7^. Soil enzymes are natural catalysts for many soil processes involved in decomposing organic matter, and participate in the processes of releasing and producing plant nutrients^8–11^. Therefore, measuring enzymatic activity provides an early indicator of changes in the intensity of biological processes and the level of soil degradation, and usually correlates with its physical and chemical properties.

Monitoring studies on the impact of transport infrastructure on the soil environment may expand the knowledge that is required to implement appropriate pro-ecological solutions, to reduce degradation (of existing facilities) and to preserve natural values (of facilities being designed).

One of the areas exposed to pollution by exhaust fumes is 830 hectares recreation area. It is one of the biggest city parks in Poland. A busy main road runs adjacent to the park.

The present research aimed to determine the impact of automobile transport on the contents of selected heavy metals in soils from a park area in the immediate vicinity of a busy main road, and to determine the relationship between their contents and enzymatic activity. We hypothesised that: (1) soil physicochemical properties would be significantly deteriorated and the content of some heavy metals would be increased by the impact of traffic, and (2) the activity of some enzymes in soil would be reduced under the influence of anthropogenic disturbance.

## Materials and Methods

### Study area

**Figure 1:**
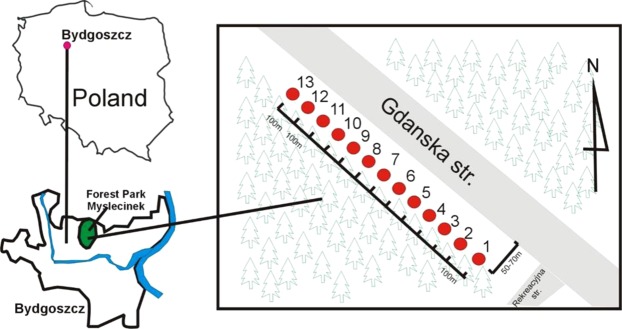
Investigated area (Author H. Jaworska).

The research was carried out in a forest park with an area of 830 ha, including 470 ha of wooded area (Fig. 1). It is the largest city park in Poland, and is located at the geographical coordinates 53°15′N, 18°03′E, in central Poland, Europe. The park’s stand consists mainly of pine forest, mixed forest and broadleaf forest. The area is located in a transitional temperate climate zone. The park is located to the north of the city of Bydgoszcz, bordering Gdańska Street to the east, while along the south-west side there runs a railway line, and a park bypass road to the north. The year-average temperature is 8.4 °C, and the year-average precipitation is 533 mm. Precipitation is lowest in February, with an average of 24 mm, and the most occurs in July (on average 78 mm). With a mean temperature of 18.3 °C, July is the hottest month of the year.

### Sample collection and preparation

The research was carried within the park, in the immediate vicinity of the road Gdańska Street is 7.3 km long, including a 3.8-km section north of the rail viaduct that has a completely different character, because it runs through a little-urbanised area, i.e. the area of the Forest Park of Culture and Recreation. The material included 26 soil samples, classified as Halic Podzol (USDA 2012)^12^, collected from two depths of 0–20 cm and 20–40 cm from 13 research points located 50 m from the road. The distance between research points was 100 m (Fig. 1). A total of 26 samples were carefully stored in clean plastic vessels before processing and analysis, and were labelled with the sampling location, date and number. In the laboratory, the soil samples were air-dried in a controlled clean environment. The soil samples were sieved with a ø 2-mm sieve and then ground with an agate mortar.

### Reagents

All chemicals were of analytical grade, as very low concentrations of trace metals were required for this study. Double-deionised water from a Millipore system was used to prepare solutions and dilutions.

### Sample analysis

In the research material, the following selected physicochemical properties were determined: texture (by laser diffraction method using a Mastersizer 2000 with the HydroμP; soil pH in the extracts: H_2_O and 1 M KCl 1:2.5 ratio; content of total organic carbon (TOC) (by VarioMAX CN ISO 10694 (1995). The content of available phosphorus (AP) was determined with the Egner–Riehm method – DL^13^. The total content of heavy metals was measured using atomic absorption spectroscopy using a SOLAR 969 (Unicam) after digestion in a mixture of HF and HClO_4_ acids using Crock’s and Severson’s method^14^. All analyses were conducted in three replicates and the validation of the results was based on the certified materials (reference soil sample TILL-3 and SO-4; Canada Centre for Mineral and Energy Technology). Based on the analytical results, contamination factor (*CF*) was calculated to assess the anthropogenic impact on the soil.

1 $$CF=\frac{{c}_{0-1}}{{C}_{n}}$$

where: C_0–1_ – metal content, C_n_ – geochemical background^15^; where: *CF* < 1 – low contamination factor, 1 ≤ *CF* < 3 – moderate contamination factor, 3 ≤ *CF* < 6 – significant contamination factor, *CF* ≥ 6 – very high contamination factor^16^.

In the moist soil samples of the activities of catalase, dehydrogenases, and alkaline and acid phosphatase were determined. The activity of oxidoreductase enzymes, namely dehydrogenase activity (DEH), was determined according to Thalmann method^17^. The activity of catalase (CAT) (E.C. 1.11.1.6) in soil was determined with the Johnson and Temple method^2^.

The activity of selected hydrolase enzymes: alkaline phosphatase (E.C. 3.1.3.1) (AlP) and acid phosphatase (E.C. 3.1.3.2) (AcP), with the method of Tabatabai and Bremner^18^.

Based on the enzymatic activities of the samples, the biological index of fertility (*BIF*) was calculated according to Stefanica *et al*.^19^:

2 $$BIF=\frac{1.5DEH+100kCAT}{2},$$

where: k is a proportionality factor equal to 0.01.

The indices of biochemical soil activity (*BA12*)^20,21^ were proposed based on the activities of soil enzymes, the content of clay and the content of organic carbon:

3 $$BA12=lo{g}_{10}TOC\sqrt{DEH+CAT+AlP+AcP}$$

### Statistical analysis

Pearson’s correlation coefficient (p < 0.05) was used to explore relationships between the total content of analysed metals and soil parameters and the physico-chemical parameters and the activity of some enzymes. Basic statistics were used to study tendencies (mean) and the variability (standard deviation SD, coefficient of variation CV, minimum, maximum and median) of the sample population. Principal components analysis (PCA) was adopted to assist the interpretation of elemental data.This method allows identifying the different of heavy metals, study parameters and enzymes in soil that correlate and thus can be considered as having a similar behavior and common origin (for example, natural or anthropogenic). In analysis (PCA) was applied using data for soil alkaline and acid phosphatase, catalase and dehydrogenase activities, grain size composition, pH in H_2_O and pH in KCl and the content of OC, AP, and the total of the selected heavy metals (Ni, Pb, Cd, Zn and Cu). The first three principal components (PC1, PC2 and PC3) were selected for further interpretation of the results. Cluster analysis was to reveal similarity or dissimilarity between sampling sites depending on the physical, chemical, biochemical variables. The coefficient of variation (CV) was also calculated for the parameters analysed for the entire study area. As for the values, 0–15%, 16–35%, and >36% indicate low, moderate, or high variation, respectively^22^. It was checked if the data came from a population with normal distribution by applying Shapiro-Wilk’s test (significance level, = 0.05).

Statistical analysis of the results was calculated in Statistica 12.0 for Windows Pl software.

## Results and Discussion

### Texture

Soil samples taken from the area covered by the study had a different granulometric composition (Table 1). The texture of analysed soils was sands and loamy sands (USDA)^12^. The content of sand fractions ranged from 52.6 to 93.4%, silt from 5.6 to 43.4% and clay from 1.0 to 4.0%.

**Table 1:** Texture and physicochemical properties of studied soils.

No	Depth	Texture %			Corg	pH	pH
	cm	2.0-0.05	0.05-0.002	<0.002	g kg^−1^	H_2_O	KCl
		mm	mm	mm			
1	0–20	77.2	20.8	2.0	13.8	7.7	7.5
	20–40	85.3	13.7	1.0	6.9	8	7.8
2	0–20	68.7	28.3	3.0	13.2	7.7	7.5
	20–40	71.8	26.2	2.0	12.6	7.8	7.5
3	0–20	52.6	43.4	4.0	10.1	7.3	6.8
	20–40	75.5	22.5	2.0	8.4	7.9	7.6
4	0–20	55.7	40.3	4.0	7.7	7.1	6.4
	20–40	88.9	10.1	1.0	3.6	7.6	7.6
5	0–20	81.3	17.7	1.0	11.7	6.7	6.1
	20–40	91.2	7.8	1.0	3.7	7.5	7.1
6	0–20	80.8	17.2	2.0	9.9	6.4	5.2
	20–40	77.8	20.2	2.0	6.7	7.7	7.5
7	0–20	83.1	15.9	2.0	8.3	6.7	6.0
	20–40	90.2	8.8	1.0	3.5	6.9	6.5
8	0–20	79.7	18.3	2.0	8.8	6.4	5.3
	20–40	88.9	10.1	1.0	3.6	6.9	6.3
9	0–20	78.6	18.4	3.0	9.7	6.8	6.2
	20–40	87.5	9.5	3.0	5.2	7.1	6.7
10	0–20	70.4	28.6	1.0	7.5	7.3	7.0
	20–40	86.7	12.3	1.0	4.3	6.9	6.4
11	0–20	77.6	21.4	1.0	12.3	7.1	6.9
	20–40	83.2	15.8	1.0	6.5	6.8	6.3
12	0–20	69.7	29.3	1.0	10.7	7.3	7.1
	20–40	76.7	21.3	2.0	5.2	7	6.4
13	0–20	80.1	18.9	1.0	9.8	7.5	7.1
	20–40	93.4	5.6	1.0	3.7	6.9	6.2

### Physicochemical properties

In the analysed samples, pH_H2O_ ranged from 6.4 to 7.7, and pH_KCl_ from 3.8 to 6.9 in the surface horizon, compared to pH_H2O_ from 6.2 to 7.5, and pH_KCl_ from 4.0 to 5.5 in the subsurface horizon. In soil samples, higher pH values were found in 5 surface horizons and in 8 subsurface horizons.

The content of organic carbon (OC) ranged from 7.5 to 13.5 g kg^−1^ in surface samples and from 3.5 to 12.6 g kg^−1^ in subsurface samples (Table 1).

**Figure 2:**
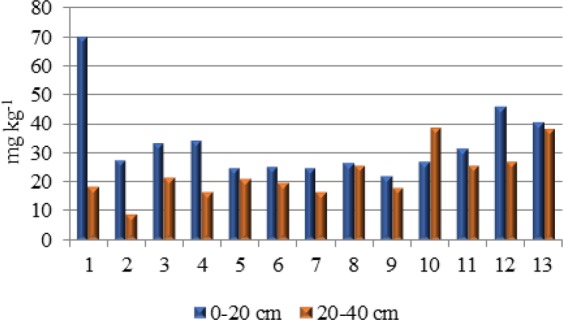
The content of available phosphorus (AP) in soil.

The content of available phosphorus in the soil ranged from 22.2 to 69.6 mg kg^−1^ (average 33.3 mg kg^−1^ in the 0–20 cm layer) (Fig. 2). In the 20–40 cm layer, the AP content on average 32% lower (from 8.9 to 38.6 mg kg^−1^). The AP content in the studied soil classifies it as having average phosphorus content (class IV) according to PN-R-04023^23^. There were no significant correlations between the phosphorus and heavy metal contents of the soil in this study. Usually, the presence of phosphorus in the soil is a significant factor in limiting the uptake of heavy metals by plants, because high levels of its more readily soluble forms can cause less soluble phosphates of zinc, cadmium, lead and copper to precipitate.

In many studies elevated heavy metal concentrations were observed in roadside soils in regional investigations^24–26^. By contrast, in the acidic soils of a forest substrate, heavy metal forms are easily absorbed by the root system^27,28^.

In the studied soils, the total content of elements in surface horizons was, in order of decreasing concentrations: Pb>Zn>Cu>Ni>Cd in subsurface horizons, compared to Zn>Pb>Cu>Ni>Cd, respectively (Table 2). These elements were clearly more abundant in surface levels, which may result from the higher humus content, but it may also be related to the impact of road traffic^27^. According to Wang *et al*.^29^ soil properties such as pH and organic matter were critical factors influencing the retention of the heavy metals seen in the studied soils.

**Table 2:** The content of selected elements.

No	Depth	Ni	Pb	Cd	Zn	Cu
	cm	mg·kg^−1^				
1	0–20	5.7	44.4	0.48	30.9	19.1
	20–40	2.4	13.0	0.00	17.9	10.1
2	0–20	3.0	20.8	0.49	26.6	11.1
	20–40	2.8	20.8	0.49	19.9	5.9
3	0–20	3.5	23.9	0.34	32.9	9.1
	20–40	2.7	18.3	0.00	19.3	3.9
4	0–20	5.1	29.6	0.45	25.1	13.8
	20–40	2.9	9.9	0.15	15.4	8.9
5	0–20	4.0	18.2	0.18	25.8	9.8
	20–40	4.0	13.4	0.00	17.8	4.1
6	0–20	7.3	24.3	0.64	19.7	21.8
	20–40	3.6	10.8	0.15	19.0	9.9
7	0–20	6.4	23.3	0.51	30.9	18.9
	20–40	5.9	13.9	0.14	26.5	10.1
8	0–20	5.4	15.9	0.36	25.1	17.7
	20–40	4.1	11.9	0.00	19.0	11.2
9	0–20	4.9	21.1	0.48	32.9	11.3
	20–40	3.2	10.4	0.18	25.2	9.7
10	0–20	5.4	34.1	0.49	25.1	12.3
	20–40	5.0	19.4	0.00	20.2	4.1
11	0–20	6.2	28.9	0.35	43.1	19.9
	20–40	5.1	16.8	0.14	23.9	19.1
12	0–20	5.5	36.3	0.44	30.9	20.1
	20–40	4.9	15.7	0.18	21.2	14.5
13	0–20	6.3	29.5	0.35	32.9	19.1
	20–40	5.2	15.2	0.00	22.1	12.0

The potential for heavy metal accumulation in the topsoil decreases as distance from roads increases, and it is assumed that after exceeding a distance of 150 m their content stabilises at a level typical of ecosystems not exposed to the impact of transport and communication^30^. In the studied soils, lead predominated in surface layers, which may be due to the proximity of the road and the quite limited solubility of minerals and their low environmental mobility^31^. Regions with higher cadmium content occur only regionally, and mainly in south-western voivodeships^31^. Unlike cadmium and lead, zinc, which predominated in the subsurface samples, participates in the metabolic processes of plants and animals and has high bioavailability^32^. Zinc also plays a significant biochemical role in the body. The highest level of pollution accumulation was recorded within 70 m of the roadside^26^.

Taking into account the permissible content of heavy metals in soil as defined in the Regulation of the Minister of Environment (of Poland) of 1 September 2016 on methods for assessing pollution of the earth’s surface^33^, the examined soils should be considered unpolluted by trace elements. Furthermore, the study area can be classified as uncontaminated according to EU values^34–36^. The determined pollution index for Ni, Pb, Cd, Zn and Cu (Table 3) is higher in all surface layer samples, which suggests the effect of road traffic on the content of heavy metals in nearby soils. Specifically, this index ranges: from 1.0 to 1.8 for Ni, indicating moderate contamination with this element; from 2.7 to 5.8 for Pb, indicating significant contamination of Pb; from 2.3 to 4.3 for Cd, i.e. significant and moderate Cd contamination; from 1.1 to 2.8 for Zn, which is moderate contamination; and from 2.8 to 5.5 for Cu, i.e. significant and moderate contamination (Table 2).

**Table 3:** Contamination factor CF.

No	Depth	Ni	Pb	Cd	Zn	Cu
	cm					
1	0–20	1.4	5.8	3.2	1.7	4.7
	20–40	0.6	1.7	0.0	0.99	2.5
2	0–20	0.8	2.7	3.2	1.5	2.8
	20–40	0.7	2.7	3.3	1.1	1.5
3	0–20	0.9	3.1	2.3	1.8	2.3
	20–40	0.7	2.4	0.0	1.1	0.96
4	0–20	1.3	3.8	3.0	1.4	3.5
	20–40	0.7	1.3	1.0	0.85	2.2
5	0–20	1.0	2.4	1.2	1.4	2.4
	20–40	1.0	1.7	0.0	0.98	1.0
6	0–20	1.8	3.2	4.3	1.1	5.5
	20–40	0.9	1.4	1.0	1.0	2.5
7	0–20	1.6	3.0	3.4	1.7	4.7
	20–40	1.5	1.8	0.0	1.5	2.5
8	0–20	1.3	2.1	2.4	1.4	4.4
	20–40	1.0	1.6	0.0	1.0	2.8
9	0–20	1.2	2.7	3.2	1.8	2.8
	20–40	0.8	1.4	1.2	1.4	2.4
10	0–20	1.4	4.4	3.3	1.4	3.1
	20–40	1.3	2.5	0.0	1.1	1.0
11	0–20	1.6	3.8	2.3	2.4	4.9
	20–40	1.3	2.2	0.9	1.3	4.8
12	0–20	1.4	4.7	2.9	1.7	5.2
	20–40	1.2	2.0	1.2	1.2	3.6
13	0–20	1.6	3.8	2.3	1.8	4.8
	20–40	1.3	1.9	0.0	1.2	3.0

Heavy metals contaminating the soil inhibit the growth of microorganisms, disrupting their basic physiological functions, and primarily processes related to the decomposition and transformation of organic matter.

### The activity of some enzymes in soil

Changes in the soil enzymes depending on the place of sampling are given in Table 4. Catalase activity ranged from 0.076 to 0.100 mg H_2_O_2_ kg^−1^ h^−1^ (average 0.087 mg H_2_O_2_ kg^−1^ h^−1^), and was higher than the subsurface layer (0.062–0.089 mg H_2_O_2_ kg^−1^ h^−1^). The lowering of enzyme activity deeper into the soil profile is related to the spatial distribution of humus and soil microorganisms and the decreasing amount of carbon substrates available to microorganisms and enzymes. Based on the calculated coefficient of variation CV (8.37% and 9.74%), the homogeneity of the results for this enzyme activity was found to be high (Table 5).

**Table 4:** The activity of catalase, dehydrogenases, alkaline and acid phosphatase in soil.

No	Depth	CAT	DEH	AlP	AcP
	cm				
1	0–20	0.095	0.158	1.912	2.590
	20–40	0.087	0.129	0.688	1.569
2	0–20	0.100	0.164	2.075	2.545
	20–40	0.090	0.153	1.337	2.611
3	0–20	0.088	0.155	1.534	2.406
	20–40	0.082	0.142	0.191	0.485
4	0–20	0.083	0.150	1.695	2.123
	20–40	0.068	0.127	0.373	0.841
5	0–20	0.087	0.115	1.171	2.337
	20–40	0.080	0.075	0.506	1.098
6	0–20	0.086	0.123	1.212	2.340
	20–40	0.076	0.079	0.327	0.540
7	0–20	0.087	0.127	1.340	2.562
	20–40	0.063	0.077	0.490	0.681
8	0–20	0.082	0.112	1.346	1.868
	20–40	0.076	0.070	0.450	1.286
9	0–20	0.080	0.092	1.080	1.384
	20–40	0.073	0.075	0.882	1.024
10	0–20	0.088	0.128	1.694	2.039
	20–40	0.082	0.073	0.808	1.618
11	0–20	0.100	0.120	1.273	2.958
	20–40	0.083	0.125	1.952	2.187
12	0–20	0.076	0.097	0.672	1.580
	20–40	0.088	0.142	1.125	2.226
13	0–20	0.081	0.117	0.700	1.855
	20–40	0.079	0.117	0.741	2.199

CAT – catalase [mg H_2_O_2_ kg^−1^ h^−1^], DEH - dehydrogenases [mg TPF kg^−1^ 24 h^−1^], AlP– alkaline phosphatase [mMpNP kg^−1^ h^−1^], AcP – acid phosphatase [mMpNP kg^−1^ h^−1^].

**Table 5:** Statistical parameters of the selected soil properties.

Parameters	Depth	Min	Max	Mean	Median	SD	CV %
	cm						
AlP	0–20	0.672	2.075	1.362	1.340	0.420	30.88
	20–40	0.191	1.951	0.759	0.688	0.483	63.63
AcP	0–20	1.384	2.957	2.199	2.337	0.444	20.20
	20–40	0.484	2.611	1.412	1.286	0.715	50.62
DEH	0–20	0.092	0.164	0.127	0.123	0.022	17.86
	20–40	0.069	0.152	0.106	0.117	0.031	29.89
CAT	0––20	0.076	0.100	0.087	0.086	0.007	8.376
	20–40	0.062	0.089	0.079	0.079	0.008	9.742
2.0-0.05	0–20	52.61	83.10	73.50	77.60	9.770	95.46
	20-40	71.80	93.40	84.39	86.70	6.827	46.61
0.05–0.002	0–20	15.90	43.40	24.50	20.80	8.967	80.41
	20-40	5.60	26.2	14.14	13.30	6.49	42.12
<0.002	0–20	1.00	4.00	2.076	2.00	1.115	1.24
	20–40	1.00	3.00	1.46	1.00	0.660	0.44
Corg	0–20	7.5	13.8	10.26	9.90	2.009	4.04
	20–40	3.5	12.6	5.68	5.20	2.616	6.85
pH H_2_O	0–20	6.4	7.7	7.09	7.10	0.446	0,19
	20–40	6.79	8.03	7.31	7.10	0.430	0.19
pH KCl	0–20	5.23	7.49	6.55	6.76	0.740	0.55
	20–40	6.20	7.78	6.89	6.67	0.582	0.34
Ni	0–20	3.04	7.30	5.29	5.41	1.204	1.45
	20–40	2.36	5.92	3.99	4.03	1.154	1.33
Pb	0–20	15.99	44.41	26.97	24.28	7.956	63.3
	20–40	9.91	20.81	14.58	13.57	3.506	12.29
Cd	0–20	0.18	0.64	0.43	0.45	0.112	0.01
	20–40	0.00	0.49	0.11	0.14	0.139	0.019
Zn	0–20	19.65	43.11	29.37	30.87	5.809	33.75
	20–40	15.40	26.45	20.55	19.98	3.136	9.83
Cu	0–20	9.05	21.80	15.73	17.65	4.591	21.08
	20–40	3.85	19.01	9.48	9.98	4.376	19.15
AP	0–20	22.17	69.59	33.29	27.37	2.841	164.89
	20–40	8.85	38.57	22.75	21.25	8.376	70.15

SD standard deviation, CV [%] - coefficient of variation.

In the soil samples taken from the top layer, the dehydrogenase activity ranged from 0.092 to 0.164 mg TPF kg^−1^ 24 h^−1^ (mean 0.127 mg TPF kg^−1^ 24 h^−1^) and was higher than in the 20–40 cm layer (mean 0.106 mg TPF kg^−1^ 24 h^−1^). In the case of dehydrogenases activity, based on the calculated coefficient of variation CV, the variance of the results was determined to be average (17.86% and 29.89%) (Table 5). Analysis of the distribution showed that most DEH activity results in the 0–20 layer are below average, as confirmed by the median value being below the mean (Table 5). The highest activity of both oxidoreductive enzymes was found in soil samples taken from point 2 (0–20 cm) (Table 4). This may be related to the OC content being higher here than at the other research points.

The activity of AlP was in the range 0.672–2.075 mM PNP kg^−1^ h^−1^ (mean 1.362 mM PNP kg^−1^ h^−1^) in the 0–20 cm layer, and from 0.191 to 1.951 mM PNP kg^−1^ h^−1^ (mean 0.725 mM PNP kg^−1^ h^−1^) in the 20–40 cm horizon (Table 4). The majority of AlP activity results were below the mean value in both tested horizons. The AcP activity in soil samples ranged from 1.384 to 2.957 mM PNP kg^−1^ h^−1^ (mean 2.199 mM PNP kg^−1^ h^−1^) from 0–20 cm, and from 0.484 to 2.611 mM PNP kg^−1^ h^−1^ (mean 1.412 mM PNP kg^−1^ h^−1^) in the 20–40 cm horizon. Alkaline phosphatase activity was on average 38% lower than acid phosphatase activity in the surface layer, and 25% lower than the same in the subsurface layer. The calculated CV was higher in soil samples from the 20–40 cm layer.

**Figure 3:**
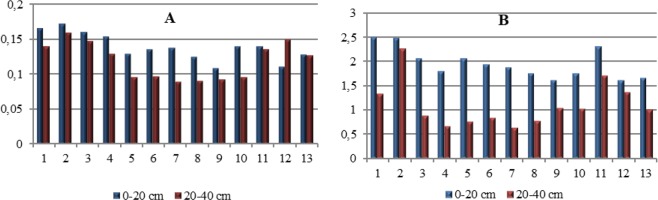
(**A**,**B**) Indices for study enzymes: (**A**) BIF, (**B**) BA1.

The higher *BIF* values^19^ were observed in the surface layer (Fig. 3A). The lowest *BA12* values were found in soil samples taken from points 5–9 (Fig. 3B). Wyszkowska *et al*.^20^ found that the activity of this indicator depend mostly on the activity of DEH and the content of OC.

### Statistical analysis

Based on the analysis of Pearson’s straight line correlation, there were significant positive relationships between the content of OC in the soil and AlP activity (*r* = 0.571; *P* = 0.0023), AcP (*r* = 0.615; *P* = 0.0008), DEH (*r* = 0.585; *P* = 0.0017) and CAT (*r* = 0.721; *P* = 0.0003) (Table 6). Similar results in earlier studies were also obtained by Gülser and Erdoğan^37^ and Bartkowiak *et al*.^8^. Other measures of soil enzymes dependence on OC and heavy metal contents were the coefficient of determination (R^2^) and the regression equation. Based on the calculated R^2^ coefficient, it was found that OC accounted for 32.62%, 37.8%, 34.3% and 52.1%, respectively for the activity of the tested enzymes. Linear regression equations show that a 1.0 g kg^−1^ increase in organic carbon content in soil saw an increase in activity of alkaline phosphatase by 0.0943 mM pNP kg^−1^h^−1^, of acid phosphatase by 0.133 mM pNP kg^−1^h^−1^, of dehydrogenases by 0.0052 mg TPF kg^−1^ 24 h^−1^ and of catalase by 0.0019 mg H_2_O_2_ kg^−1^ h^−1^. Organic matter has a protective function for enzymes, which are immobilised by it. It positively affects the stabilisation of protein structures, reducing sensitivity to negative changes caused by environmental factors. Analysis of the correlations showed weak, however significant positive relationships between Pb content and the activity of AlP (*r* = 0.519; *P* = 0.0065), AcP (*r* = 0.537; *P* = 0.0046), DEH (*r* = 0.432; *P* = 0.0271) and CAT (*r* = 0.503; *P* = 0.0090), and between Cd and the tested enzymes: AlP (*r* = 0.638; *P* = 0.0004), AcP (*r* = 0.569; *P* = 0.0024), DEH (*r* = 0.4602; *P* = 0.0179) and CAT (*r* = 0.444; *P* = 0.0230) (Table 6). This is probably related to the beneficial physicochemical properties of the studied soils having reduced the mobility of heavy metals, at the same time reducing their toxicity to enzyme proteins. Organic matter and clay fraction bind enzyme protein, protecting it against negative environmental factors. It is also known that, in low concentrations, heavy metals are activators for many enzymes. It should also be emphasised that in the conducted tests, no permissible heavy metal levels were exceeded, which indicates their natural content in the soil, which also did not inhibit the investigated oxidoreductive or hydrolytic enzymes. By contrast, Gülser and Erdoğan^37^ obtained significant negative correlations with the enzymes studied (arylsulfatase, acid phosphatase and urease). The effect of heavy metals varies depending on soil enzymes^37^. Cadmium can inhibit dehydrogenase, catalase and urease, while zinc can only inhibit catalase and urease, and lead inhibits catalase and urease to a lesser extent than Cd or Zn^20^. These authors have shown that the synergistic effect of Cd, Zn and Pb results in a greater inactivation of enzyme activity than the same metals in isolation. Changes in soil enzymatic activity also depend on hydrothermal conditions – temperature distribution and rainfall – as the factors most affecting microorganisms and the associated enzymatic activity of soil^38^. Significant positive relationships were also found between the content of lead, zinc and cadmium and OC content (Table 6). Lead is an element that is not very mobile in soils and has low availability for plants. However, it can form various complex organic and inorganic compounds that are relatively easily absorbed by plant roots and accumulated. A similar relationship was identified for zinc. Zinc is highly mobile and usually accumulates in the surface layers of mineral soils and in humus by bonding with organic matter. Meanwhile, cadmium accumulates fastest and most abundantly - of all metals, in organic horizons. It can also easily enter the food chain, due to its good solubility and bioavailability (Alloway and Ayres 1999). An important factor in increasing the toxicity of heavy metals in soils is pH. In the studied soils, a statistically significant negative correlation was found between nickel content and pH (Table 6). In soils of pH < 5.0, phytotoxicity clearly increases^31^.

**Table 6:** Relationship between selected soil properties.

Variables		Regression equation	*r*	*R*^*2*^	*P*
AlP	Corg	AlP = 0.308 + 0.0943_Corg_	0.571	0.326	0.0023
AcP	Corg	AcP = 0.7434 + 0.133_Corg_	0.615	0.378	0.0008
DEH	Corg	DEH = 0.0753 + 0.0052_Corg_	0.585	0.343	0.0017
CAT	Corg	CAT = 0.0682 + 0.0019_Corg_	0.721	0.521	0.0003
AlP	Pb	AlP = 11.860 + 8.4048_Pb_	0.519	0.270	0.0065
AcP	Pb	AcP = 8.8034 + 6.629_Pb_	0.537	0.289	0.0046
DEH	Pb	DEH = 5.6214 + 129.57_Pb_	0.432	0.187	0.0271
CAT	Pb	CAT = −22.3489 + 518.5_Pb_	0.503	0.253	0.0090
AlP	Cd	AlP = 0.0129 + 0.2413 _Cd_	0.638	0.408	0.0004
AcP	Cd	AcP = −0.0274 + 0.164 _Cd_	0.569	0.324	0.0024
DEH	Cd	DEH = −0.1077 + 3.2195 _Cd_	0.460	0.212	0.0179
CAT	Cd	CAT = −06197 + 10.683 _Cd_	0.444	0.197	0.0230
AlP	Zn	AlP = 19.827 + 4.843_Zn_	0.407	0.166	0.0388
AcP	Zn	AcP = 16.951 + 4.437 _Zn_	0.489	0.239	0.0111
CAT	Zn	CAT = 0.3145 + 296.4_Zn_	0.391	0.153	0.0479
AlP	Cu	AlP = 8.2395 + 4.1176_Cu_	0.409	0.167	0.0377
AcP	Cu	AcP = 5.6685 + 3.8422_Cu_	0.501	0.251	0.0091
AlP	Hg	AlP = 0.0217 + 0.0243_Hg_	0.663	0.439	0.0002
AcP	Hg	AcP = 0.0256 + 0.0121_Hg_	0.433	0.187	0.0271
Zn	Corg	Zn = 15.975 + 1.1268_Corg_	0.574	0.329	0.0022
Pb	Corg	Pb = 6.7239 + 1.7615_Corg_	0.659	0.435	0.0002
Cd	Corg <	Cd = −0.0847 + 0.0443_Cd_	0.710	0.505	0.0005
Cd	<0.002	Cd = 1.2252 + 2.0236_<0.002_	0.434	0.188	0.0276
Ni	pH H_2_O	Ni = 8.1701 − 0.2081_pHH2O_	−0.625	0.392	0.0006
Ni	pH KCl	Ni = 8.1312 − 0.303_pH KCl_	−0.598	0.357	0.0012

Trace elements in soil are generally associated with soil management, while organic matter contents and their availability to plants depends on soil reaction and soil sorption capacity.

**Figure 4:**
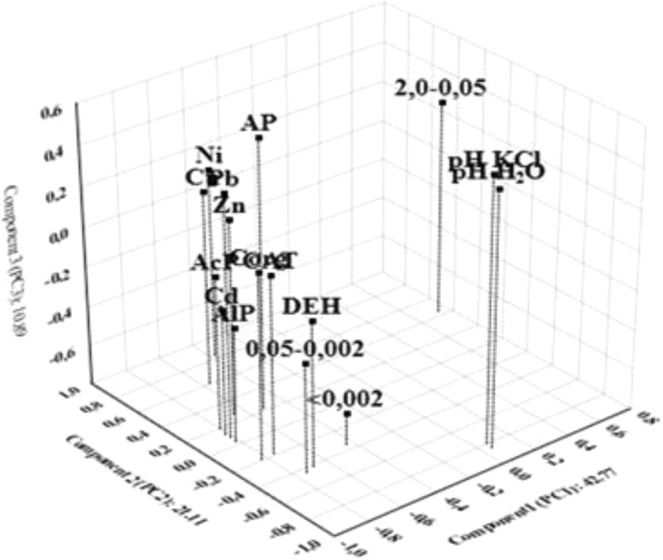
Component plot in rotated space for studied elements.

To determine the direction and strength of relations between the activity of the tested enzymes on the one hand and, on the other, selected physicochemical soil parameters (granulometric composition, pH in H_2_O and KCl, OC content, total Ni, Pb, Cd, Zn, Cu and available P) and environmental variables on the other, multivariate Principal Component Analysis (PCA) was used. Three principal components (*PC1*, *PC2* and *PC3*) extracted from the available dataset accounted for 74.77% of the variance (Fig. 4). Because the test results were expressed in various different units, the principal components were calculated using a correlation matrix.

The *PC1* component was responsible for 42.77% of all elementary variables and indicates a significant negative correlation with the activity of the alkaline phosphatase (−0.812), acid phosphatase (−0.805), dehydrogenases (−0.692) and catalase (−0.752) (Table 7). These four biochemical parameters may reflect anthropogenic pollution caused by the impact of road traffic. Significant negative correlations were also found with OC (−0.823) and the heavy metals Pb (−0.815), Cd (−0.814) and Zn (−0.662). It means that, respectively, 67.7%, 66.4% and 66.2%, 43,8% of the variance of those variables is accounted for with *PC1*. This association strongly suggests that these variables have a similar (anthropogenic) source. *PC2* accounts for 21.11% of the data variance. It has shown a significant negative relationship with pH in H_2_O (−0.849), pH in KCl (−0.806) and positive with the content of total Ni (0.837) and Cu (0.605). The third principal component (*PC3*) accounts for 10.89% of the data variation and significantly negatively correlated only with <0.002 mm (−0.633).

**Table 7:** Values of the three extracted factor loadings (*PC1, PC2 and PC3)* for 16elements.

Elements	PC1	PC2	PC3
AlP	**−0.812**	0.035	**−**0.219
AcP	**−0.805**	0.187	**−**0.024
DEH	**−0.692**	**−**0.423	**−**0.060
CAT	**−0.752**	**−**0.183	0.097
2.0-0.05	**0.733**	0.446	0.286
0.05-0.002	**−0.741**	**−**0.436	**−**0.243
<0.002	**−**0.444	**−**0.348	**−0.633**
Corg	**−0.823**	**−**0.189	0.132
pH H_2_O	0.054	**−0.849**	0.481
pH KCl	0.055	**−0.806**	0.537
Ni	**−**0.380	**0.839**	0.153
Pb	**−0.815**	0.078	0.419
Cd	**−0.814**	0.129	**−**0.165
Zn	**−0.662**	0.261	0.186
Cu	**−**0.575	**0.605**	0.183
AP	**−**0.524	0.214	0.551
Variation%	42.77	21.11	10.89

Bold values are statistically significant.

**Figure 5:**
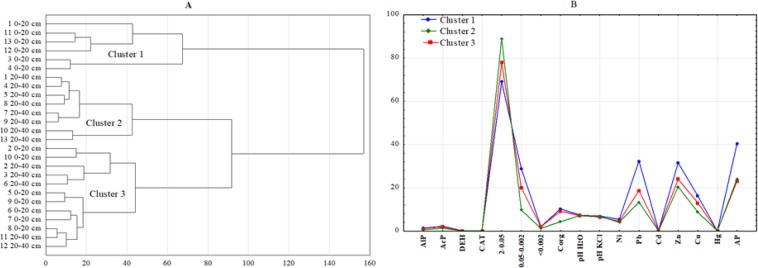
(**A**,**B**) Similarity dendrogram for sampling sites (**A**) and a graph k − means for the study properties (**B**).

In order to determine the similarities between the 13 research points (two depths), Ward’s grouping method (1963) was used. This was based on soil properties (granulometric composition, pH, organic matter content and the tested heavy metals) and the activity of the selected enzymes. The grouping procedure identified three clusters of soils with similar physicochemical and enzymatic properties (Fig. 5A). Cluster 1 groups the soils collected from points 1, 3, 4, 11, 12, 13 (0–20 cm) with high contents of AP, Zn, Cu, Pb, Ni, OC, 0.05–0.002 and the highest activity of AlP and AcP (Fig. 5B). Cluster 2 contained soils from points 1, 4, 5, 7, 8, 9, 10, 13 (20–40 cm). The soil from these places contains the most 2–0.05, the lowest OC, Pb, Zn, Cu and the lowest activity of AlP, AcP and DEH. Meanwhile, cluster 3 groups soils from points 2, 5, 6, 7, 8, 9 and 10 (0–20 cm) and 2, 3, 6, 11, 12 (20–40 cm). The activity of the tested enzymes in the soil samples in this cluster is lower than for cluster 1 but higher than for cluster 2.

## Conclusion

The soils of the studied area can be classified as unpolluted by trace elements according to permissible heavy metal contents in soil as defined in the Regulation of the Minister of Environment (of Poland) **(**Dz. U. 2016. poz. 1395) and according to EU norms^34–36^. However, the determined pollution index for Ni, Pb, Cd, Zn and Cu is higher in all surface layer samples, which confirms the impact of road traffic on the content of heavy metals in nearby soils. Correspondingly, this index falls in the following ranges: 1.0–1.8 for Ni, indicating moderate Ni contamination; 2.7–5.8 for Pb, indicating significant Pb contamination; 2.3–4.3 for Cd, i.e. significant and moderate Cd contamination; 1.1–2.8 for Zn, which is moderate Zn contamination; and 2.8–5.5 for Cu, i.e. significant and moderate Cu contamination, which indicates the need to monitor the studied area. No negative relationships were determined between the activity of the studied soil enzymes and the content of heavy metals in the soil affected by road traffic, which was related to the protective function of organic matter.

The study showed an anthropogenic source of tested heavy metals in soil, however their content did not exceed the permissible standards. The applied statistical analyzes (PCA, CA and correlation) showed that the heavy metals tested form separate groups of different origin. Two groups were distinguished: Pb, Cd and Zn - elements of anthropogenic origin and Ni and Cu of natural origin.

Due to the agrotechnical category of tested soils (light soils) and slightly acidic pH, the investigated area requires constant monitoring. The physicochemical properties of these soils causes the increase of bioavailability of heavy metals. The justification for continuing the research is the specificity of the studied area, which is recreation area. Monitoring and assessment of the degree of threat of the impact of road traffic on the soil environment will allow to introduce the environmental protection standards in the design and exploitation of communication infrastructure.
